# Single Cell RNA Transcriptomics of Mantle Cell Lymphoma Reveals the Presence of Treatment‐Resistant Subclones at the Time of Diagnosis

**DOI:** 10.1002/ajh.70270

**Published:** 2026-03-09

**Authors:** Dmitry Manakov, Magdalena Klanova, Michal Kolar, Robert Ivanek, Florian Geier, Julien Roux, Tomas Zikmund, Lucie Winkowska, Eva Kriegova, Jirina Manakova, Liliana Tuskova, Silvia Spanikova, Alexandra Scasna, Marek Trneny, Pavel Klener

**Affiliations:** ^1^ Institute of Pathological Physiology, First Faculty of Medicine Charles University Prague Czech Republic; ^2^ First Department of Medicine‐Department of Hematology Charles University General Hospital in Prague Prague Czech Republic; ^3^ Institute of Molecular Genetics, Czech Academy of Sciences Prague Czech Republic; ^4^ Department of Biomedicine, DBM Bioinformatics Core Facility University of Basel Basel Switzerland; ^5^ DBM Bioinformatics Core Facility Swiss Institute of Bioinformatics Basel Switzerland; ^6^ CLIP – Childhood Leukaemia Investigation Prague, Department of Pediatric Haematology and Oncology, Second Faculty of Medicine Charles University and University Hospital Motol Prague Czech Republic; ^7^ Department of Immunology Palacký University Olomouc and University Hospital Olomouc Olomouc Czech Republic

**Keywords:** clonality, CNV, MCL, scRNA‐seq

## Abstract

Mantle cell lymphoma (MCL) is a B‐cell malignancy with a chronically relapsing clinical course and pronounced genetic heterogeneity. To investigate the clonal dynamics underlying early disease relapse, we performed single‐cell RNA sequencing of paired tumor samples collected at diagnosis and at first relapse. Inference of copy number variants (CNVs) identified multiple subclonal clusters at both time points. In all cases, a minor subclone present at diagnosis harbored a CNV profile highly concordant with that of the dominant relapse clone, indicating the pre‐existence of therapy‐resistant subclones at diagnosis. Gene set enrichment analysis comparing therapy‐resistant and therapy‐sensitive clones revealed substantial inter‐patient heterogeneity in resistance‐associated transcriptional programs. Despite this heterogeneity, convergent dysregulation of cell‐cycle control pathways emerged as a shared feature across patients. Furthermore, we investigated a case of SOX11‐negative indolent MCL (iMCL) with late relapse characterized by extensive extranodal dissemination, including peripheral blood leukemization and intestinal tumor masses. While bone marrow‐ and peripheral blood‐derived MCL cells maintained SOX11 negativity and a largely conserved transcriptomic state, intestinal MCL cells displayed blastoid morphology, expression of SOX11, a profoundly remodeled CNV landscape, and the acquisition of multiple additional driver mutations. Collectively, these findings indicate that early relapse in MCL originates from minor therapy‐resistant subclones present at diagnosis and subsequently selected under therapeutic pressure. Moreover, disease progression in iMCL may be driven by spatially restricted clonal evolution, with the emergence of aggressive molecular features in distinct anatomical compartments. These results provide mechanistic insight into MCL clonal evolution and relapse biology.

## 1 Introduction

Mantle cell lymphoma (MCL) is a mature B cell non‐Hodgkin lymphoma characterized by a chronically relapsing clinical course [1]. Despite recent advances in therapeutic strategies, including Bruton tyrosine kinase inhibitors (BTKi) and adoptive immunotherapy with genetically engineered chimeric antigen receptor T‐lymphocytes (CAR T‐cells), immunochemotherapy remains the standard backbone of care for the majority of newly diagnosed MCL patients who require treatment [2, 3, 4]. Clonal evolution of MCL cells following failure of standard immunochemotherapy approaches remains incompletely understood [5, 6].

Genetic heterogeneity is a defining feature of MCL [7]. In addition to the canonical *IGH‐CCND1* t(11;14) translocation, numerous recurrent genetic alterations have been described, several of which were associated with poor outcome in the context of immunochemotherapy including mutations of *TP53*, *NOTCH1*, *KMT2D*, or deletion of *CDKN2A* [8, 9, 10, 11, 12].

In recent years, numerous studies implementing single cell RNA sequencing (scRNA‐seq) have been increasingly applied to investigate intratumoral heterogeneity in lymphoid malignancies [13, 14, 15]. To date, only five studies have employed scRNA‐seq in MCL [16, 17, 18, 19, 20] and of these, only one analyzed paired samples obtained at diagnosis and at relapse, with a mean progression of disease (POD) of 3.8 years.

In the present study, we used scRNA‐seq to characterize (sub)clonal evolution of MCL cells obtained from patients with MCL at diagnosis and at first early clinical relapse (POD < 12 months) following failure of standard immunochemotherapy regimens. In addition, we report the first molecularly characterized case of transformation from indolent MCL to blastoid disease.

## 2 Materials and Methods

### 2.1 Patients

MCL cells were isolated from various tissues of five patients with MCL at diagnosis and at the time of first clinical relapse following failure of standard immunochemotherapy based on alternation of R(ituximab)‐CHOP and R‐high‐dose‐araC. All patients signed informed consent in accordance with the Declaration of Helsinki. The study was approved by the Ethics Committee of the General University Hospital in Prague (approval No. 60/20), and all patients consented to the use of their biological material for the purpose of this study. Four patients were diagnosed with classical nodal aggressive MCL, all of whom experienced early relapse (i.e., POD < 12 months). One patient was diagnosed with non‐nodal iMCL and relapsed 8.5 years after therapy initiation. Progression‐free survival (PFS) was defined as time from diagnosis to first clinical relapse or progression. The best objective response during induction therapy was at least partial remission in all analyzed patients. Additional patient characteristics are provided in Supplemental Table 1.

### 2.2 Tissue Sample Handling, Cell Sorting and Single‐Cell RNA Sequencing

Patient samples were collected from peripheral blood, bone marrow, lymph node, tonsil, malignant ascites, or infiltrated intestine. Bone marrow samples were collected using trephine biopsy. Peripheral blood mononuclear cells (PBMC) and bone marrow mononuclear cells were isolated by density gradient centrifugation using Ficoll‐Paque PLUS (Cytiva). Lymph nodes, tonsils, and intestinal samples were surgically excised, mechanically homogenized at 4°C, and washed in phosphate‐buffered saline (PBS). The resulting single‐cell suspensions were cryopreserved in fetal bovine serum (FBS, BioTech a.s.) supplemented with 10% dimethyl sulfoxide (DMSO) and stored in liquid nitrogen.

Subsequent sample processing followed the protocol for frozen PBMC for scRNA‐seq (CG00039 Rev. D, 10× Genomics). Cells were thawed, centrifuged (300 × g for 5 min at 4°C), and resuspended in PBS supplemented with bovine serum albumin (PBS + BSA). A total of 2 × 10^6^ cells per 100 μL were incubated with an antibody cocktail (Supplemental Table 2, EXBIO Praha a.s.) for 30 min on ice in the dark. Cells were diluted with 800 μL PBS + BSA, filtered through a 100 μm cell strainer, and labeled with 2 μL Hoechst 33258 to exclude dead cells. Up to 150 000 cells CD45^+^ cells were sorted on an Influx cell sorter (BD Biosciences) using a 100 μm nozzle into RPMI medium supplemented with 10% FBS. Sorted cells were counted using a TC20 cell counter (Bio‐Rad) to assess viability and subsequently loaded into individual Chromium GEM wells. Target and recovered cell numbers for each sample are listed in Supplemental Table 3. Libraries were prepared using the Chromium Next GEM Single Cell 3′ protocol, reagent kits v3.1 with dual indexing (10× Genomics). Final libraries were pooled at equimolar concentrations and sequenced in paired‐end mode (150 bp) on an Illumina NovaSeq 6000 platform.

### 2.3 scRNA‐Seq Data Processing and Analysis

Raw sequencing reads were aligned to the human reference genome GRCh38 provided by 10× genomics (version GRCh32‐2020‐A, GENCODE v32/Ensembl 98) and gene expression was quantified using STARsolo (v2.7.10b) [21] according to the Chromium Single Cell 3′ v3.1 protocol specifications. UMI deduplication and cell barcode matching were performed allowing one mismatch. Gene expression counts were derived from uniquely mapped exonic reads.

Cell barcodes were identified using emptyDropsCellRanger (false discovery rate [FDR] < 0.01, DropletUtils v1.26.0 [22]). Ambient RNA contamination was removed using decontX (v1.4.1) [23], with the empty droplets matrix used as ambient background; the contamination threshold was set to < 0.9. Doublets were identified and removed using scDblFinder (v1.20.2) with default clustering [24]. Thresholds for transcript counts, UMI counts, and the percentage of mitochondrial genes (“^MT‐”) were defined individually for each sample to identify low‐quality cells (Supplemental Table 3). Samples were initially clustered as described below, and clusters containing > 33% low‐quality cells were excluded from downstream analyses.

Cells passing quality control (QC) were merged and log‐normalized using Seurat (v5.3.0). Highly variable genes (HVGs) were identified using variance‐stabilizing transformation (VST), followed by linear scaling and principal component analysis (PCA). Uniform manifold approximation and projection (UMAP) was performed using principal components explaining at least 1.5% of the variance. Clustering was conducted using the Leiden algorithm based on the selected principal components. No data integration or batch‐correction was applied.

Clusters were annotated based on canonical marker gene expression, including B cells (*MS4A1*, *CD79B*, *IGHM*), CD8+ T cells (*CD3G*, *CD8A*), CD4+ T cells (*CD3G*, *CD4*), NK cells (*NKG7*, *GNLY*), monocytes (*CD14*, *LYZ*, *FCN1*). Clusters were further subset by tissue of origin to annotate rarer cell populations, including plasma cells (*JCHAIN*) and B cell progenitors in bone marrow samples (*IGLL1*, *PAX5*). Malignant cells were identified in per‐sample subsets based on uniformly high CCND1 expression (> 1 log count in 93.6% of cells on average) and the ratio of *IGLC* or *IGKC* expression. Malignant cells in P069 (iMCL) were further subset and re‐clustered to distinguish them from normal B cells.

Cell‐cycle phases were inferred using the CellCycleScoring function in Seurat, and phase distributions between diagnostic and relapse samples were compared using the chi‐square test with Benjamini‐Hochberg correction.

Copy number variations were inferred in paired diagnostic/relapse samples using inferCNV (v1.21.0) [25] with default settings, including a gene expression cutoff of 0.1, a six‐state hidden Markov Model, and Ward. D2 hierarchical clustering. The tumor subcluster *p*‐value threshold was set to 0.01. Cells were clustered using Leiden's algorithm.

Pairwise comparisons between therapy‐resistant and therapy‐sensitive subclones at diagnosis were performed using the Wilcoxon rank‐sum test (minimum log fold change of 0.25 in at least 10% of cells). Genes with an adjusted *p*‐value < 0.05, absolute log_2_ fold change > 0.8, and expression in > 25% of cells were considered significant. Gene set enrichment analysis of Gene Ontology (GO) terms and Hallmark gene sets was performed using clusterProfiler (v4.14.6) on log_2_ fold change‐ranked genes with an absolute log_2_ fold change > 0.25, applying Benjamini‐Hochberg correction and *p*‐value threshold of < 0.05.

### 2.4 Whole Exome Sequencing

Genomic DNA was extracted from fresh frozen cells using the DNeasy Blood & Tissue Kit (Qiagen) according to the manufacturer's protocol. Germline DNA was obtained from either buccal swabs or bone marrow samples without detectable lymphoma infiltration by flow cytometry. All tumor tissue samples included in whole‐exome sequencing (WES) analysis contained ≥ 30% tumor cells as assessed by flow cytometry.

Sequencing libraries were prepared using the SureSelectXT Human All Exon V6 + UTR kit (Agilent) and sequenced on an Illumina NextSeq 500 instrument. Sequencing reads were aligned to the human reference genome hg38 using BWA‐MEM [26] and processed according to the GATK workflow (v4.6.1.0) [27]. Somatic variants were identified using Mutect2 [28] and annotated with Funcotator using GENCODE and HGNC databases (v1.8.hg38).

Copy number alterations were inferred using CNVkit (v0.9.11) [29]. Target regions were defined according to the capture regions of the library preparation kit. For each tumor sample, copy number ratios were calculated by normalizing coverage against a sex‐matched pooled reference generated from normal samples.

To assess correlations between CNV profiles, genomic segments were uniformly binned across autosomal chromosomes using 100 kb bins. For bins overlapping multiple segments, copy number values were averaged. Values were normalized by subtracting the diploid baseline (copy number = 2), and Spearman's rank correlations were calculated.

### 2.5 Optical Genome Mapping

Frozen cell pellets were processed using the Bionano Prep SP Frozen Cell Pellet DNA Isolation kit (Bionano Genomics, San Diego, CA, USA). High‐molecular weight DNA was labeled using the Bionano Prep Direct Label and Stain DLS DNA Kit (Bionano Genomics) according to the manufacturer's instructions and analyzed on the Bionano Saphyr platform to generate approximately 250× optical mapping coverage per genome, as described previously [30]. Data were analyzed using Bionano Access (v1.5) and the Bionano Solve (v3.5) software, applying bioinformatics pipelines for both *de novo* and rare variants detection, alignment to the hg38 reference genome, filtering of somatic structural variants, and merging of results according to the manufacturer's instructions.

## 3 Results

### 3.1 Single‐Cell RNA Sequencing of MCL Cells Obtained From Different Compartments at Diagnosis and at Relapse

Single cell RNA sequencing was performed on five paired samples obtained from patients with MCL at diagnosis and at first clinical relapse following failure of standard immunochemotherapy. The best response to induction therapy was complete remission in three patients and partial remission in two patients. Four patients (P009, P022, P027, P087) were diagnosed with conventional nodal MCL, and all experienced an early relapse (POD < 12 months). Among these, patient P009 had a rare t(11;22) translocation, which was confirmed by optical genome mapping (Supplemental Figure 1, Supplemental Table 4). Patient P069 was diagnosed with indolent, non‐nodal, SOX11‐negative MCL and relapsed 8.5 years after initiation of therapy with aggressive disease; results for this patient are therefore presented separately.

**FIGURE 1:**
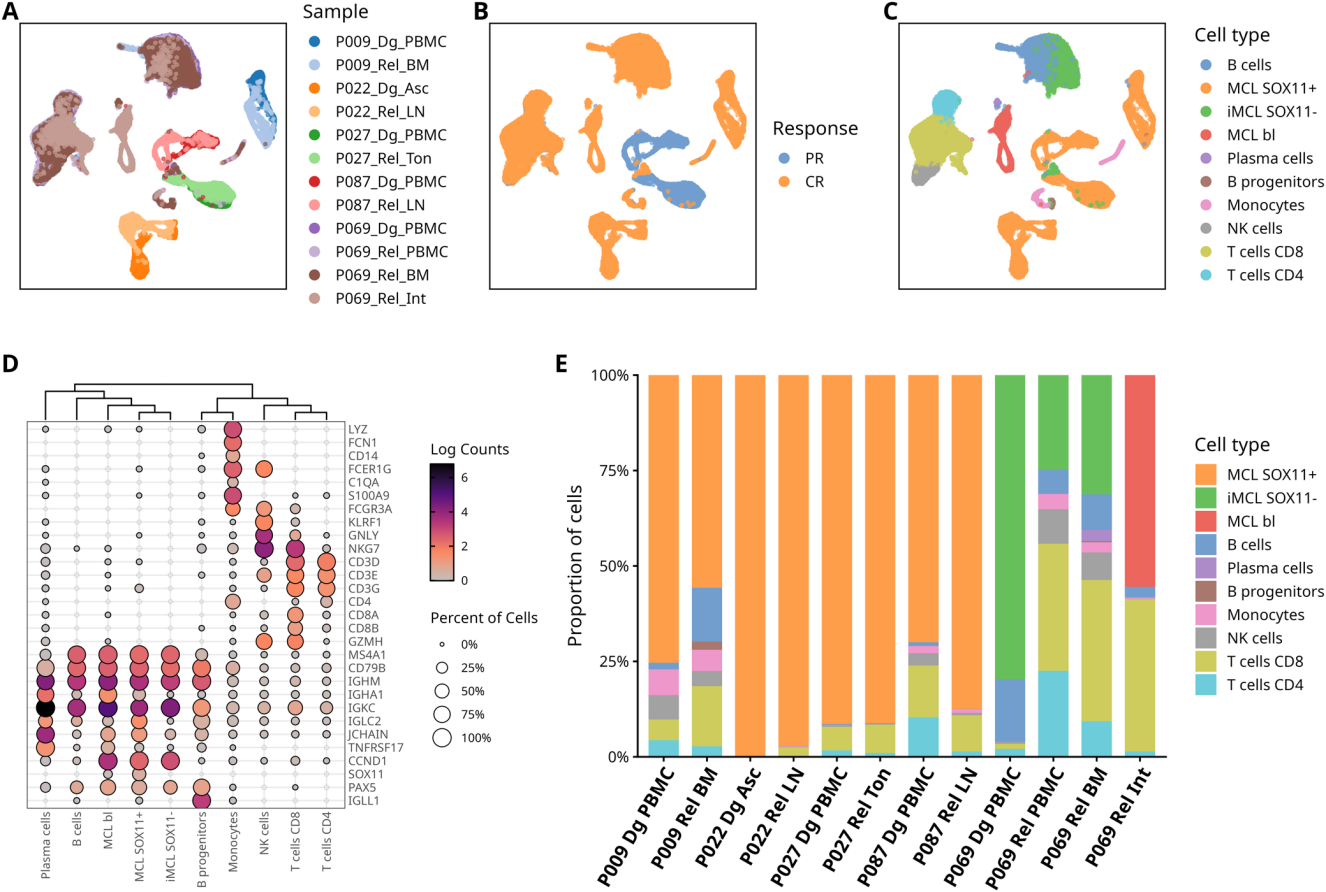
Single‐cell RNA sequencing dataset overview and cell type annotation. UMAP projection of 58 702 cells colored by sample origin (A), therapy response (PR—partial remission, CR—complete remission), (B) and cell type (C). Each patient was sampled at diagnosis (Dg) and at relapse (Rel) timepoints from various tissue compartments (PBMC—peripheral blood mononuclear cells, BM—bone marrow, ASC—ascites fluid, LN—lymph node, TON—tonsil, GUT—intestinal tissue). (D) Dot plot showing expression of marker genes across identified cell types. Circle size represents the percentage of cells expressing each gene within the cell type, while color intensity indicates average log‐transformed expression levels. Genes are arranged to highlight key markers used for cell type identification. (E) Per‐sample proportions of each cell type. [Color figure can be viewed at wileyonlinelibrary.com]

A total of 58 702 cells passed quality control, with a median of 1547 genes detected per cell (Figure 1A,B). Unsupervised, sample‐specific clustering revealed a diverse cellular composition, of which 40 069 cells were identified as MCL cells (68.2%, Figure 1C–E). The remaining cells were classified as non‐malignant, including CD8+ T cells (14.2%), CD4+ T cells (5.4%), B cells (7.5%), NK cells (2.6%), monocytes (1.5%) and B cell progenitors (0.13%). Despite originating from different patients and tissues, non‐malignant cells formed distinct shared clusters in the merged dataset, whereas MCL cells largely retained sample‐specific clustering patterns. The only exception comprised iMCL cells from patient P069, which resembled normal B cells but were separable upon subset re‐clustering. Consistent with clinical annotation, MCL cells from patients with aggressive disease expressed *CCND1* and *SOX11* (Supplemental Figure 2). Tumor cell clusters from patient P009 exhibited lower *CCND1* expression compared with those from the other nodal MCL patients (mean log expression 1.0 vs. 2.74), most plausibly attributable to the rare t(11;22) translocation. In addition, higher average *SOX11* expression was observed in tumor cell clusters from patient P009 compared to the remaining nodal MCL patients (mean log expression 0.94 vs. 0.42).

Comparison of diagnostic and relapse MCL cells across the four patients with nodal disease (P009, P022, P027, P087) revealed extensive transcriptional remodeling during disease progression. On average, 586 were upregulated and 274 genes were downregulated at relapse (Supplemental Table 5). Patient P087 exhibited the most pronounced transcriptional changes (1304 upregulated, 241 downregulated genes), whereas patient P027 showed the most modest alterations (119 upregulated and 177 downregulated genes). Three genes—*NUDT1* (nudix hydrolase 1), HMGA1, and HMGB3 (high‐mobility group proteins)—were consistently upregulated at relapse across all four patients.

Analysis of cell‐cycle phase distribution demonstrated a shift from G1 phase toward the S and G2/M phases at relapse (chi‐square test, *p* < 0.01; Supplemental Table 6 and Supplemental Figure 3). Patient P009 exhibited the most pronounced shift, with a 45% decrease in G1‐phase cells and corresponding increases in proliferative phases (S: +11%, G2/M: +34%).

### 3.2 Single‐Cell CNV Clonotyping Reveals Small Diagnostic Clones With Transcriptome Profiles Similar to the Major Relapse Clone

Copy number inference identified four to seven distinct cellular clusters (hereafter referred to as subclones, Supplemental Table 7) within each paired sample. Based on longitudinal changes in subclone abundance between diagnosis and relapse, subclones were classified as either “**therapy‐resistant”** (expanded or persistent at relapse) or “**therapy‐sensitive”** (depleted or reduced at relapse).

**FIGURE 2:**
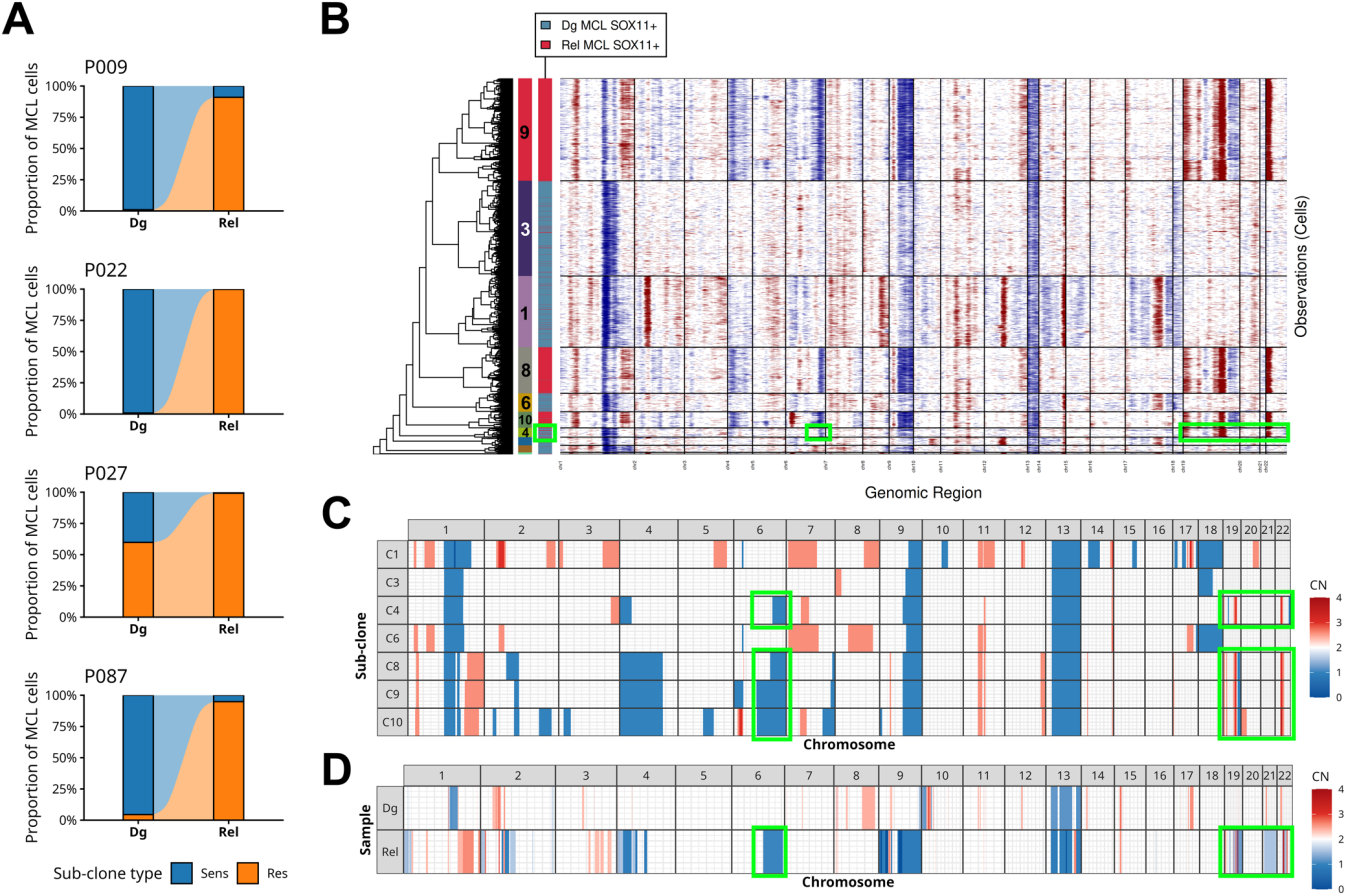
Copy number variation‐based subclonal analysis reveals therapy‐driven clonal evolution. (A) Proportions of “therapy‐sensitive” (Sens) and “therapy‐resistant” (Res) cells at diagnosis (Dg) and relapse (Rel) in four aggressive MCL patients. (B) Joint clustering of inferred copy number patterns in diagnosis (Dg) and relapse (Rel) MCL cells of patient P087. Rows of the heatmap represent individual tumor cells clustered by CNV similarity, with subclone assignments indicated by numbered bars on the left. Columns represent genomic regions ordered by chromosomal position. Red indicates copy number gains, blue indicates losses, and white represents diploid regions. Sample origin is indicated by the right annotation bar. Green rectangles highlight the subclone detected at diagnosis that carries CNVs specific for subclones at relapse (del 6q, amp 19p, 19q, 22p). (C) Averages of copy numbers inferred from scRNA‐seq data per each sub‐clonal cluster. Green rectangles highlight the CNVs specific for “therapy‐resistant” subclones. (D) Copy numbers predicted using WES data from Dg and Rel samples. Green rectangles highlight the CNVs specific for “therapy‐resistant” subclones detected in Rel. [Color figure can be viewed at wileyonlinelibrary.com]

In patients P009, P022, and P027, cells corresponding to the dominant relapse subclones were detectable already at diagnosis as small therapy‐resistant subclones (Figure 2A, Supplemental Figures 4–6). These therapy‐resistant subclones accounted for 1%, 0.8%, and 59.8% of the respective diagnostic MCL populations of patients P009, P022, and P027 respectively, and were substantially larger in patient P027, who achieved partial remission, compared with patients P009 and P022, who achieved complete remission. In patient P087, diagnostic subclone C4 (4.3% of the diagnostic MCL population) shared a characteristic pattern of amplifications on chromosomes 19 and 22 with the relapse‐dominant subclones C8, C9, and C10 (Figure 2B,C, Supplemental Figure 7). The presence of these amplifications was independently confirmed using WES (Figure 2D). These findings suggest that diagnostic subclone C4 represented a therapy‐resistant precursor of relapse despite not clustering directly with the relapse‐specific subclones.

Comparison of CNV segments inferred by inferCNV with those detected using CNVkit from WES‐based bulk data showed significant positive correlations across all patient samples. Spearman's correlation coefficients ranged from 0.29 in patient P022 to 0.57 in patient P027 at diagnosis, and from 0.24 in patient P009 to 0.65 in patient P022 at relapse, with a mean ρ of 0.47 (Supplemental Table 8).

### 3.3 Genomic Landscape of Therapy‐Resistant Subclones

We next explored genomic alterations inferred from CNV profiles in individual subclones (Supplemental Table 9).

In patient P009, deletions of 1p22.3‐1p21.2, 17p13.3‐17q11.2, 19p13.3, and 22q12.2‐13.1, as well as amplifications of 14q32.13‐32.33 and 22q11.21‐12.1, were shared across all subclones. Notably, the 17p deletion encompassed *TP53* and *HIC1*. Both the therapy‐sensitive subclone C3 and therapy‐resistant subclones C4/C5 harbored a deletion of 9p24.1‐21.1 involving *CDKN2A* and *CDKN2B*, together with amplifications of 22p13.2 (comprising *EP300*) and 22p13.31.

All subclones from patient P022 shared deletions of 11q22.2‐25 (comprising *CHEK1*) and 17p13.3‐p11.2 (comprising *TP53*), as well as amplifications of 3q11.2‐29 (comprising *PIK3CA/B*, *BCL6*) and 18q12.1‐q23 (comprising *BCL2*). The resistant subclones harbored an additional amplification of 2q24.2‐37.3, encompassing *NFE2L2* (NRF2).

Patient P027 exhibited the least complex CNV landscape among the nodal MCL cases, with amplifications of 6p25.3‐21.2 (comprising *E2F3*) and 17q22‐25.1 (comprising *PRKCA*), and a deletion of 6q16.3‐6q27 (encompassing *ARID1B*, *TNFAIP3, FOXO3*) present in all subclones. In contrast, amplification of 8q12.1.13‐24.3 encompassing *MYC* and *PVT1* genes was restricted to therapy‐sensitive subclones, whereas therapy‐resistant populations lacked this alteration.

All subclones from patient P087 shared deletions of 1p13.1‐1q24.2, 9q22.31‐9q34.3, and 13q12‐13.34 (encompassing *RB1*, *BRCA2* and the *DLEU1/DLEU2* locus), as well as an amplification of 14q32.32‐32.33. Diagnosis subclone C4 and relapse subclones C8, C9, and C10 shared additional deletions of 4p16.3‐14, 6q22.31‐27 (encompassing *TNFAIP3*, *PRKN*), and 9q21.13‐34.3 (encompassing *TSC1, PTCH1, NOTCH1*), together with amplifications of 19p13.3, 19q13.11‐13.32 (encompassing *AKT2*, *CD79A*, *BCL3*), and 22q11.1‐12.1.

Subclone‐specific CNVs were further validated by intersecting inferCNV‐derived segments with WES‐derived copy number alterations from matched diagnosis or relapse samples, retaining only concordant gains or losses (Supplemental Figure 8, Supplemental Table 10).

### 3.4 Differential Gene Expression Analysis Reveals Patient‐Specific Resistance Mechanisms

**FIGURE 3:**
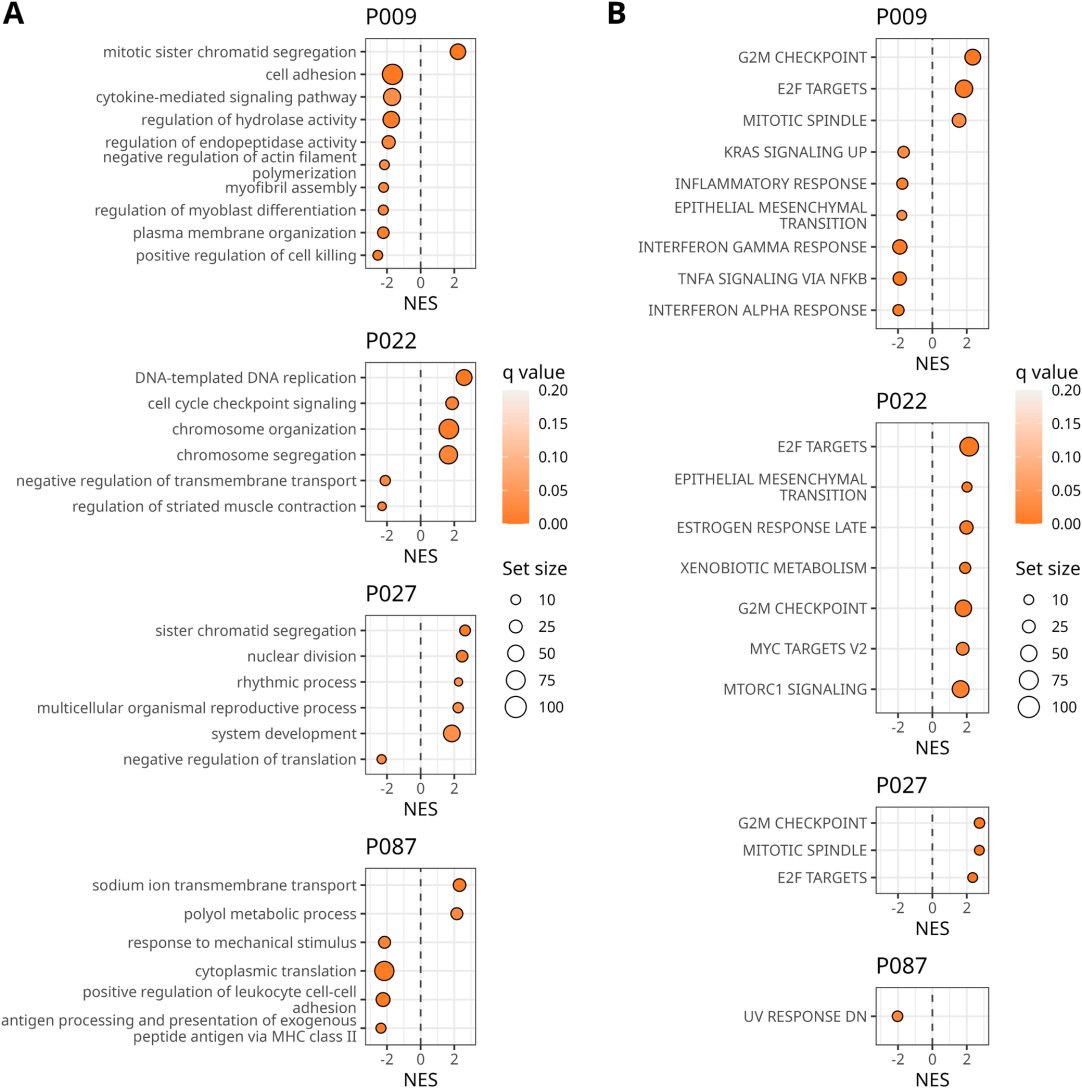
Differential expression‐based comparison of therapy‐resistant to therapy‐sensitive subclone cells at diagnosis. (A) Gene set enrichment of Gene Ontologies terms. (B) Analysis of Hallmark gene sets enrichment. NES—normalized enrichment score. [Color figure can be viewed at wileyonlinelibrary.com]

Comparative analysis of therapy‐resistant versus therapy‐sensitive subclones at diagnosis revealed enrichment of the G2/M checkpoint and E2F targets Hallmark gene sets, as well as the chromosome segregation Gene Ontology term, in patients P009, P022, and P027 (Figure 3, Supplemental Tables 11, 12). In contrast, patient P087 showed reduced enrichment of Gene Ontology terms related to MHC class II expression. The number of significantly differentially expressed genes in therapy‐resistant subclones at diagnosis was highest in P087 (286 genes), compared with 2, 10, and 27 genes in patients P009, P022, and P027, respectively (Supplemental Table 13).

### 3.5 Patient P069—A Molecular Characterization of Indolent to Blastoid MCL Transformation

Patient P069 was unique in several respects. First, P069 was the only indolent, non‐nodal MCL patient who achieved a long‐term remission after induction immunochemotherapy. Second, relapse samples were available from three distinct anatomical compartments (leukemic peripheral blood, infiltrated bone marrow, and infiltrated intestine) in contrast to diagnostic leukemic peripheral blood only. Third, at relapse, immunohistochemistry revealed an aggressive, SOX11‐positive, blastoid MCL variant in the involved intestine, associated with a rapidly progressive clinical course.

**FIGURE 4:**
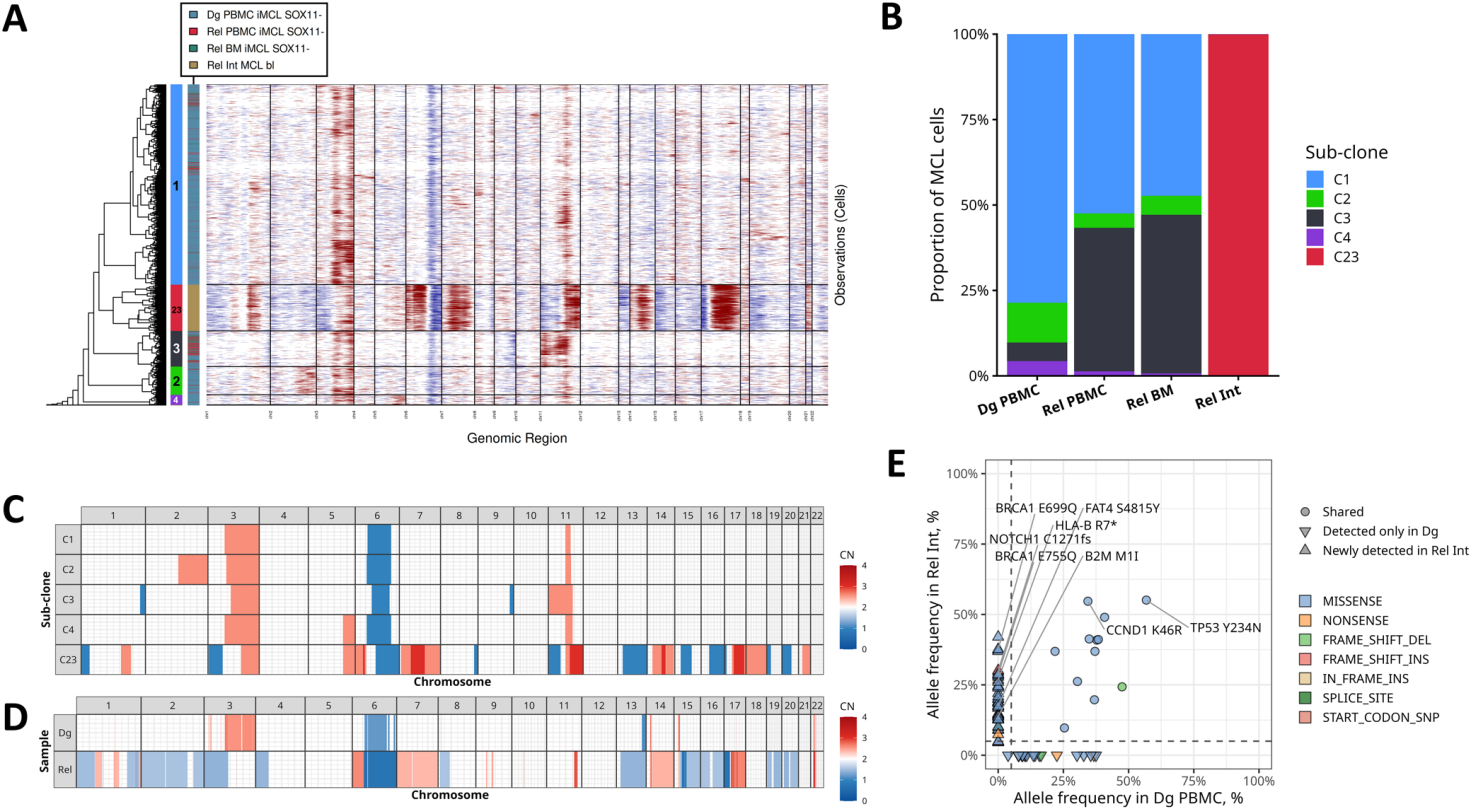
Copy number variations and somatic mutations in indolent MCL patient P069 across diagnosis and relapse timepoints. (A) Joint clustering of inferred copy number patterns in diagnosis (Dg) and relapse (Rel) MCL cells using inferCNV. Rows represent individual tumor cells clustered by CNV similarity, with subclone assignments indicated by left annotation bar. Columns represent genomic regions ordered by chromosomal position. Red indicates copy number gains, blue indicates losses, and white represents diploid regions. Sample origin is indicated by the right annotation bar. (B) Sub‐clone proportions in each of P069 samples. The colors correspond to left bar on the inferCNV heatmap. (C) Averages of copy numbers inferred from scRNA‐seq data per each sub‐clonal cluster. (D) Copy numbers predicted using WES data from Dg and Rel samples. (E) Variant allele frequency (VAF) scatter plot comparing somatic mutations in PBMC at diagnosis versus intestine at relapse. Points are colored by mutation type and shaped by detection pattern across samples. Dashed lines indicate 5% VAF threshold. Selected high‐impact mutations are labeled. [Color figure can be viewed at wileyonlinelibrary.com]

CNV inference identified four subclonal populations present at diagnosis and in both late relapse samples (Figure 4A,B, Supplemental Figure 9). All subclones shared amplifications of 3p14.1‐3q29 and 11q13.1‐14.2, as well as a deletion of 6p12.3‐6q24.1 encompassing *PRDM1* (Figure 4C, Supplemental Table 9). At relapse, subclone C3, which harbored additional deletions of 1q42.13‐44 and 9q33.2‐34.3, and an amplification of 11p, expanded markedly from 5% of the diagnostic MCL population to 42% in peripheral blood and 46% in bone marrow (Figure 4B). Differential expression analysis comparing C3 with other diagnostic subclones revealed upregulation of *TAF10* (log fold change = 0.82 in 28% of cells).

The intestinal relapse sample contained a single dominant subclone, C23 (Figure 4A,B). In contrast to the relatively simple genomic profiles of other subclones, C23 displayed a markedly complex CNV profile with 11 deletions and 19 amplifications. Nevertheless, amplifications of 3p26.2‐3q29 and 11q13.2‐14.1, together with a deletion of 6q14.1‐23.2 were shared with the remaining subclones. Alterations unique to C23 affected multiple genomic regions harboring key oncogenes and tumor suppressors. Deleted regions included *TP53*, *RB1*, *ARID1A*, *FOXO3*, *BACH2*, *BRCA2*, and *SMARCA4*, whereas amplified regions encompassed *CDK6*, *MCL1, BCL2*, *BIRC2/3*, *STAT3/5*, and *MALT1*, among others. CNV inference for C23 was validated by WES, demonstrating strong concordance (Spearman's *ρ* = 0.63, Figure 4D).

Consistent with the scRNA‐seq findings, WES‐based analysis of protein‐coding somatic variants identified 16 mutations unique to the diagnosis sample, 48 variants newly detected in the intestinal relapse, and 12 variants shared between both time points (Figure 4E). Shared variants included missense variants in *TP53* and *CCND1*, both with variant allele frequency (VAFs) of 55%. Newly detected variants comprised a *NOTCH1* frame shift insertion (VAF = 30%), a nonsense mutation of *HLA‐B* (VAF = 26%), and two missense mutations of *BRCA1* (VAF = 37% and 29%). The median VAF of newly detected variants was 17.5%, indicating substantial subclonal heterogeneity in the transformed relapse. A complete list of single nucleotide variants (SNVs) identified in patient P069 is provided in Supplemental Table 14.

## 4 Discussion

This study builds upon our previously published WES‐based analysis of 25 patients with MCL, in which we investigated clonal evolution at diagnosis and at first clinical relapse following failure of standard front‐line immunochemotherapy [6]. In that study, we identified several novel mutations in relapsed samples, along with increased variant allele frequencies (VAFs) of *TP53*, an increased frequency of *CDKN2A* deletions, and overall increased genetic heterogeneity in relapsed lymphoma compared with diagnostic samples. These findings both confirm and extend observations reported by other groups [5, 31, 32]. Based on these results, we hypothesized that MCL relapse is driven by genetically adverse subclones already present at the time of diagnosis. This concept of pre‐existing “therapy‐resistant” clones was subsequently supported by Joffe et al. [33].

To further delineate the (sub)clonal evolution of lymphoma cells, we performed scRNA‐seq‐based analysis of primary MCL cells obtained at diagnosis and at first clinical relapse following failure of conventional immunochemotherapy.

Computational inference of CNVs from scRNA‐seq data enabled identification of small diagnostic subclones whose CNV profiles overlapped with, or closely matched, those of dominant relapse clones. These findings support the notion that such minor diagnostic subclones represent therapy‐resistant populations. By contrast, the remaining therapy‐sensitive diagnostic subclones were markedly reduced or nearly eradicated at lymphoma relapse, consistent with the clinical achievement of partial or complete remission in response to frontline therapy. This subclonal reshaping occurred within a relatively short interval between treatment initiation and disease relapse (mEFS = 6.5 months) and was consistently observed across multiple tissue compartments. These observations are concordant with previous reports of pronounced clonal evolution associated with inferior outcomes in longitudinal MCL cohorts [31].

As expected, scRNA‐seq analyses revealed pronounced subclonal heterogeneity within MCL populations both at diagnosis and at relapse in all patients, consistent with finding reported in other lymphoma subtypes [13, 34, 35]. Prior studies in MCL have demonstrated transcriptionally distinct subclones at relapse in patients treated with ibrutinib [17, 18]. Hansen et al. analyzed eight newly diagnosed MCL patients and observed minimal subclonal diversity in seven cases, while reporting substantial inter‐patient heterogeneity [16]. Notably, only one patient in that cohort died from MCL (median follow up 38.5mo), suggesting a potential association between subclonal diversity and adverse prognosis. A longitudinal study by Zhang et al. focusing on ibrutinib‐treated patients demonstrated the emergence of transcriptionally heterogeneous tumor subpopulations characterized by 17q amplification gain and upregulation of *BIRC5* (survivin) [18]. The same group later analyzed 15 patients treated with CAR T‐cells following BTK inhibitor failure, reporting decreased cytotoxic T‐cell populations and increased expression of the immune checkpoint receptor TIGIT on MCL cells after CAR T‐cell treatment [19]. More recently, Wan et al. analyzed 11 MCL patients with an average time to relapse of 4.2 years, including four patients with paired diagnostic and relapse samples, and demonstrated marked transcriptional heterogeneity already present at diagnosis [20]. Taked together, these data suggests that both baseline genomic complexity and type of treatment (i.e., chemotherapy, targeted agents, CAR T‐cells) critically shape the mechanisms of (sub)clonal selection in MCL.

Our findings extend this body of work by demonstrating the presence of small therapy‐resistant subclones in all four patients who experienced early relapse, implicating these populations as key contributors to treatment failure. Transcriptional profiling of the therapy‐resistant versus therapy‐sensitive subclones at diagnosis revealed consistent deregulation of cell‐cycle‐related programs, with enrichment of G2/M checkpoint and E2F targets Hallmark gene sets, as well as chromosome segregation‐related Gene Ontology terms, in three of four patients with aggressive MCL. In contrast, other deregulated pathways varied substantially between patients, underscoring inter‐patient heterogeneity in resistance mechanisms. This diversity highlights the importance of a single‐cell‐based approach for accurately detecting and potentially targeting genetically and transcriptionally heterogeneous therapy‐resistant MCL subpopulations.

In contrast to the four patients with early relapse, CNV profiles of MCL cells isolated from peripheral blood and bone marrow of patient P069, who was diagnosed with indolent, non‐nodal MCL, remained stable between diagnosis and relapse, which occurred more than 6 years after the last dose of rituximab maintenance therapy. Notably, MCL cells obtained from the infiltrated intestine at relapse displayed a markedly distinct CNV profile, a finding independently confirmed by WES. Immunohistochemical analysis of the intestinal infiltrate revealed blastoid variant MCL with a high proliferation index as indicated by Ki‐67. These observations suggest that patient P069 experienced an indolent relapse in the bone marrow with subsequent peripheral blood leukemization, potentially preceding blastoid transformation within the intestinal compartment by several months (or even years). This hypothesis was further supported by WES data demonstrating the acquisition of multiple driver mutations in intestinal MCL cells, including alterations in NOTCH1 previously implicated in indolent‐to‐aggressive transformation [10, 36, 37]. Blastoid transformation of MCL is rare, with only limited cases reported to date [38, 39, 40]. To our knowledge, this study provides the first detailed molecular characterization of indolent‐to‐blastoid transformation of MCL.

Several limitations of this study should be acknowledged. The relatively small patient cohort may limit the generalizability of our findings. In addition, the number of sequenced cells constrained detection sensitivity for rare resistant subclones, particularly in patients P009 and P022. CNV inference from scRNA‐seq data is inherently susceptible to discrepancies relative to DNA‐based approaches due to transcriptional deregulation and technical artifacts such as gene dropout, which may obscure true CNVs or introduce spurious calls. Moreover, changes within the tumor microenvironment (TME) may facilitate the survival and expansion of therapy‐resistant subclones while sensitive populations are eliminated, as described in another lymphoma subtypes [41]. Our study was not designed to directly interrogate TME effects, as diagnostic and relapse samples were often derived from distinct anatomical compartments. Previous analyses of the MCL microenvironment have revealed complex cell–cell interactions, including increased CD70‐CD72 signaling at late relapse [20]. Future spatially resolved single‐cell studies integrating malignant cells with their microenvironment will therefore be essential to disentangle the relative contributions of genetic and microenvironmental factors in therapy resistance.

In conclusion, our study demonstrates that scRNA‐seq can be leveraged not only to investigate disease biology and cellular heterogeneity but also to identify therapy‐resistant subclones at diagnosis prior to treatment initiation. Although relapse samples were required for identification of resistant diagnostic subclones in this proof‐of‐concept cohort, larger studies may enable prospective prediction of individual therapeutic responses at diagnosis. Single‐cell RNA sequencing thus represents a powerful approach for dissecting tumor heterogeneity and may contribute to the future development of more personalized therapeutic strategies for patients with MCL.

## Author Contributions

**Dmitry Manakov:** investigation (lead); methodology (lead); data curation (lead); formal analysis (equal); project administration (equal); resources (supporting); software (lead); validation (lead); visualization (lead); writing – original draft preparation (equal). **Magdalena Klanova:** project administration (equal), writing – original draft preparation (equal). **Michal Kolar:** resources (supporting); investigation (supporting). **Robert Ivanek:** investigation (supporting); formal analysis (equal); software (supporting). **Florian Geier:** investigation (supporting); formal analysis (equal); software (supporting). **Julien Roux:** investigation (supporting); formal analysis (equal); software (supporting). **Tomas Zikmund:** investigation (supporting); formal analysis (equal); software (supporting). **Lucie Winkowska:** resources (supporting); data curation (supporting). **Eva Kriegova:** validation (supporting); resources (supporting). **Jirina Manakova:** validation (supporting); resources (supporting). **Liliana Tuskova:** investigation (supporting); formal analysis (supporting). **Silvia Spanikova:** investigation (supporting); formal analysis (supporting); software (supporting); visualization (supporting). **Alexandra Scasna:** investigation (supporting); formal analysis (supporting); software (supporting); visualization (supporting). **Marek Trneny:** resources (supporting); funding acquisition (supporting). **Pavel Klener:** funding acquisition (lead); supervision (lead); project administration (equal); resources (lead); writing – original draft preparation (equal).

## Funding

This work was supported by Grantová Agentura České Republiky, GA23‐05474S. National Institute for Cancer Research, LX22NPO5102.

## Conflicts of Interest

The authors declare no conflicts of interest.

## Supporting information


**Data S1:** Supporting Information.

## Data Availability

Raw FASTQ files for both scRNA and whole‐exome sequencing have been deposited in the European Genome‐phenome Archive, dataset EGAD50000001213, available at https://ega‐archive.org/studies/EGAS50000000825. The processed and annotated Single Cell Experiment object is available at https://doi.org/10.5281/zenodo.13220568.
