# Supplementary material for: Single Cell RNA Transcriptomics of Mantle Cell Lymphoma Reveals the Presence of Treatment‐Resistant Subclones at the Time of Diagnosis
Source: Am J Hematol. 2026 Mar 9;101(5):1025–35. doi: 10.1002/ajh.70270 (PMC13055129; doi:10.1002/ajh.70270)
Supplement: Supplementary file 1 — Data S1: Supporting Information. [file AJH-101-1025-s001.docx]

**Supplemental Data File**

**Title: *Single Cell RNA Transcriptomics of Mantle Cell Lymphoma Reveals the Presence of Treatment-Resistant Subclones at the Time of Diagnosis.***

**Supplemental Table 1.** Patient cohort characteristics.

| **Code** | **Age** | **Sex** | **MIPI** | **Morphology** | **Ki-67 (%)** | **Therapy*** | **ASCT** | **Best response to front-line Tx**** | **EFS (months)** | **OS (months)** | **Status** |
| --- | --- | --- | --- | --- | --- | --- | --- | --- | --- | --- | --- |
| P009 | 47 | F | 7.3 | Classic | 90 | A | 1 | CR | 8 | 10 | dead |
| P022 | 63 | M | 7 | Blastoid | 100 | A | 0 | CR | 5 | 10 | dead |
| P027 | 68 | M | 8 | Pleomorphic | 90 | B | 0 | PR | 5 | 31 | dead |
| P069 | 76 | M | 7.3 | N/A | 5*** | B | 0 | CR | 102 | 107 | dead |
| P087 | 66 | M | 7.1 | Classic | 70 | A | 1 | PR | 9 | 11 | dead |

* **A** = Nordic protocol, that is, alternation of R-Maxi-CHOP and R-HDAC (2–3 g/m2, 4 doses every 12 h), 3 + 3 cycles, autologous stem cell transplant (ASCT), and rituximab maintenance, **B** = modification of CLSG-MCL1 protocol, that is, alternation of R-CHOP and R-DHAOx-senior (dexamethasone, HDAC, reduced oxaliplatin), 3 + 3 cycles, and rituximab maintenance. ** **CR** = complete remission, **PR** = partial remission. ******* assessed from the bone marrow.

**Supplemental Table 2.** Antibodies used for cell staining for FACS.

| **Population** | **Antigen** | **Fluorochrome** | **Cat.N.** | **Clone** | **Amount per sample, ul** |
| --- | --- | --- | --- | --- | --- |
| White Blood Cells | CD45 | PE | 1P-222-T100 | MEM-28 | 20 |
| B-cells | CD19 | APC | 1A-305-T100 | LT19 | 20 |
| NK-cells | CD56 | FITC | 1F-231-T100 | MEM-188 | 20 |
| Monocytes | CD14 | AF700 | A7-212-T025 | MEM-18 | 10 |
| T-cells | CD8 | PerpCy5.5 | T9-207-T025 | MEM-31 | 5 |
| T-cells | CD4 | PECy7 | T7-359-T025 | MEM-241 | 5 |

**Supplemental Table 3.** Cell sorting and quality control.

| **Patient ID** | **Time**  **point** | **Tissue***** | **Hoechst− CD45+, %*** | **CD19+, %**** | **Post-sort viability, %** | **Target cell count** | **UMI threshold** | **Genes threshold** | **Mitochondrial genes threshold, %** | **Cells past QC** | **Cells past QC, %** |
| --- | --- | --- | --- | --- | --- | --- | --- | --- | --- | --- | --- |
| P009 | Dg | PBMC | 90 | 95 | 98 | 3500 | 2500 | 1000 | 8 | 2824 | 83 |
|  | Rel | BM | 70 | 82 | 98 | 3500 | 1500 | 800 | 12 | 2914 | 83.4 |
| P022 | Dg | Asc | 71 | 99 | 99 | 5000 | 2500 | 1000 | 5 | 3017 | 84.7 |
|  | Rel | LN | 82 | 97 | 97 | 5000 | 2000 | 1000 | 5 | 3479 | 76 |
| P027 | Dg | PBMC | 96 | 90 | 96 | 5000 | 2000 | 1000 | 10 | 3359 | 92.9 |
|  | Rel | Ton | 14 | 90 | 98 | 5000 | 1000 | 300 | 8 | 3221 | 84.7 |
| P087 | Dg | PBMC | 95 | 68 | 96 | 3500 | 2000 | 1000 | 10 | 2881 | 81.4 |
|  | Rel | LN | 94 | 92 | 98 | 3500 | 1700 | 900 | 10 | 2023 | 76.8 |
| P069 | Dg**** | PBMC | 80 | 92 | 99 | 10000 | 900 | 400 | 6 | 7644 | 88.4 |
|  |  |  |  |  | 98 | 10000 | 900 | 400 | 6 | 9738 | 84.7 |
|  | Rel | PBMC | 99 | 40 | 97 | 10000 | 1800 | 600 | 15 | 7666 | 86.6 |
|  |  | BM | 99 | 50 | 94 | 10000 | 1800 | 800 | 15 | 4693 | 85.6 |
|  |  | Int | 98 | 68 | 94 | 10000 | 1000 | 600 | 20 | 5243 | 58.7 |
|  |  |  |  |  |  |  |  |  | **Total:** | **58 702** | **81.5%** |

* − percentage of Hoechst− CD45+ cells among all singlet cells in the sample, measured during FACS.

** − percentage of CD19+ cells in Hoechst− CD45+ cell population measured during FACS.

*** - PBMC – peripheral blood mononuclear cells, BM – bone marrow, LN – lymph node, Ton – tonsil, Asc – ascitic fluid, Int – intestinal tissue.

**** − DG sample from P069 was prepared in technical duplicate to mitigate the risk of microfluidic channel clogging during single-cell capture.

**Supplemental Table 4.** Results of optical genomic mapping for patient P009. The table lists genomic aberrations shared for diagnosis and relapse and those exclusive at both timepoints.

The table is available at <https://doi.org/10.5281/zenodo.13220568>

**Supplemental Table 5.** Results of differential gene expression analysis between relapse and diagnosis MCL cells of four patients (P009, P022, P027 and P087) performed using Wilcoxon rank-sum test. Only genes with adjusted p-value < 0.05, absolute log2 fold change > 1, and expression in at least 25% of relapse cells are included.

The table is available at <https://doi.org/10.5281/zenodo.13220568>

**Supplemental Table 6.** Changes in cell cycle phase distributions compared using chi-square test with Benjamini-Hochberg P value correction across four patients with aggressive MCL.

| **Patient** | **Chi squared** | **P adjusted** | **G1** | **S** | **G2M** |
| --- | --- | --- | --- | --- | --- |
| P009 | 936.0 | 2.3E-203 | -0.45 | 0.11 | 0.34 |
| P022 | 348.0 | 3.67E-76 | -0.19 | 0.18 | 0.01 |
| P027 | 16.5 | 2.65E-04 | -0.02 | 0.00 | 0.02 |
| P087 | 414.3 | 2.19E-90 | -0.31 | 0.24 | 0.08 |

**Supplemental Table 7.** Sub-clones detected using CNV inference in patients with aggressive MCL. Ratios were calculated as ratio of cells at relapse (Rel) to sum of Rel to ratio of cells at diagnosis (Dg) to sum of Dg. Direction of log2 ratio change was used to assign the subclone to either therapy-sensitive (Sens) or therapy-resistant (Res) type. * - subclone C4 in P087 was designated as Res based on copy number structure similarity to relapse subclones C8, C9 and C10.

| **Patient** | **Subclone** | **Dg** | **Rel** | **Ratio** | **Log ratio** | **Type** |
| --- | --- | --- | --- | --- | --- | --- |
| P009 | C1 | 1017 | 100 | 0.13 | -2.93 | Sens |
| P009 | C2 | 519 | 45 | 0.11 | -3.17 | Sens |
| P009 | C3 | 566 | 1 | 0.00 | -9.97 | Sens |
| P009 | C4 | 18 | 1205 | 87.62 | 6.45 | Res |
| P009 | C5 | 3 | 271 | 118.24 | 6.89 | Res |
| P022 | C1 | 2826 | 0 | 0.00 | -9.97 | Sens |
| P022 | C2 | 163 | 4 | 0.02 | -5.57 | Sens |
| P022 | C3 | 11 | 1633 | 132.34 | 7.05 | Res |
| P022 | C4 | 14 | 1744 | 111.05 | 6.80 | Res |
| P027 | C1 | 1123 | 1744 | 1.63 | 0.71 | Res |
| P027 | C2 | 1229 | 23 | 0.02 | -5.57 | Sens |
| P027 | C3 | 103 | 129 | 1.31 | 0.39 | Res |
| P027 | C4 | 605 | 1019 | 1.77 | 0.82 | Res |
| P087 | C1 | 685 | 32 | 0.05 | -4.29 | Sens |
| P087 | C3 | 910 | 49 | 0.06 | -4.04 | Sens |
| P087 | C4 | 79 | 14 | 0.19 | -2.39 | Res* |
| P087 | C6 | 177 | 9 | 0.05 | -4.29 | Sens |
| P087 | C8 | 3 | 461 | 162.42 | 7.34 | Res |
| P087 | C9 | 1 | 1030 | 1088.66 | 10.09 | Res |
| P087 | C10 | 1 | 161 | 170.17 | 7.41 | Res |

**Supplemental Table 8.** Spearman’s correlations between binned CNV regions detected using inferCNV and averaged across subclones - sensitive (Sens) or resistant (Res), and CNVkit from exome-sequenced DNA samples (Dg or Rel). All correlations are significant with p < 0.0001.

| **Patient** | **Subclones** | **WES sample** | **Spearman's rho** |
| --- | --- | --- | --- |
| P009 | Sens | Dg | 0.42 |
| P009 | Res | Rel | 0.24 |
| P022 | Sens | Dg | 0.29 |
| P022 | Res | Rel | 0.65 |
| P027 | Sens | Dg | 0.54 |
| P027 | Res | Rel | 0.54 |
| P087 | Sens | Dg | 0.38 |
| P087 | Res | Rel | 0.46 |
| P069 | Sens | Dg | 0.61 |
| P069 | Res (intestinal) | Rel (intestinal) | 0.63 |

**Supplemental Table 9. Copy number (CN) variations of the subclones detected using inferCNV**.

The table is available at <https://doi.org/10.5281/zenodo.13220568>

**Supplemental Table 10. Copy number variations of the subclones detected using inferCNV clustering and intersected with copy number variations predicted using WES data**.

The table is available at <https://doi.org/10.5281/zenodo.13220568>

**Supplemental Table 11.** Gene Set Enrichment Analysis (GSEA) of GO Biological Processes for resistant vs. sensitive subclones at diagnosis. GSEA results showing enriched GO Biological Process terms in resistant versus sensitive MCL subclones at diagnosis for each patient (P009, P022, P027, P087). Positive NES values indicate enrichment in resistant subclones. Only terms with q-value < 0.05 are shown.

The table is available at <https://doi.org/10.5281/zenodo.13220568>

**Supplemental Table 12.** Gene Set Enrichment Analysis of Hallmark pathways for resistant vs. sensitive subclones at diagnosis. GSEA results showing enriched MSigDB Hallmark pathways in resistant versus sensitive MCL subclones at diagnosis for each patient (P009, P022, P027, P087). Positive NES values indicate enrichment in resistant subclones. Only terms with q-value < 0.05 are shown.

The table is available at <https://doi.org/10.5281/zenodo.13220568>

**Supplemental Table 13.** Results of differential gene expression comparison between relapse and diagnosis MCL cells of four patients (P009, P022, P027 and P087) performed using Wilcoxon rank-sum test. Only genes with adjusted p-value < 0.05, absolute log2 fold change > 1, and expression in at least 25% of cells in the upregulated group are included.

The table is available at <https://doi.org/10.5281/zenodo.13220568>

**Supplemental Table 14. Somatic nucleotide variants detected in P069 PBMC at diagnosis and in the infiltrated intestine (Int) at relapse. The variants were called using Mutect2 and annotated using Gencode43 database.**

The table is available at <https://doi.org/10.5281/zenodo.13220568>

**Supplemental Data Object 1.** SingleCellExperiment object with 58 702 cells passed QC and 36601 genes. Data columns include sample, patient, timepoint, compartment, cell type annotation (cell_type), cell cycle phase (Phase), tumor subclones (inferCNV_subclone).

**
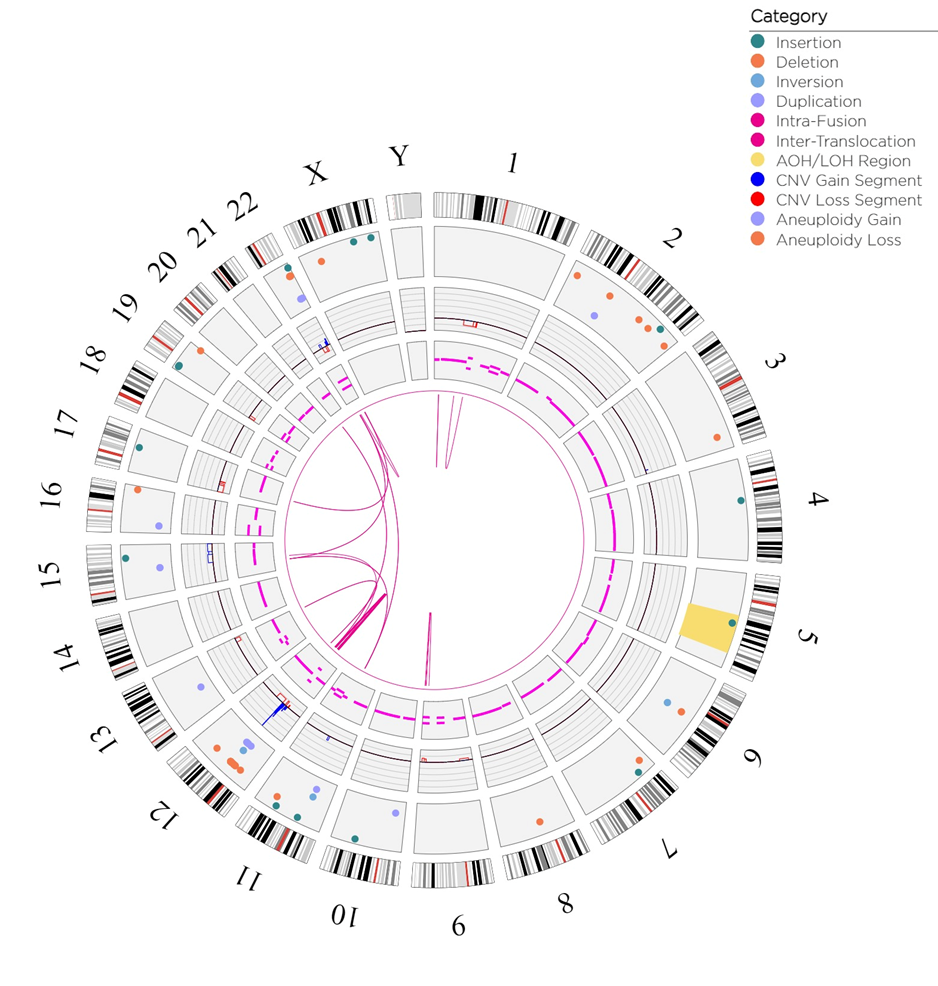
**

**Supplemental figure 1.** Optical genomic mapping results visualized as a circular genomic diagram for P009 at diagnosis.


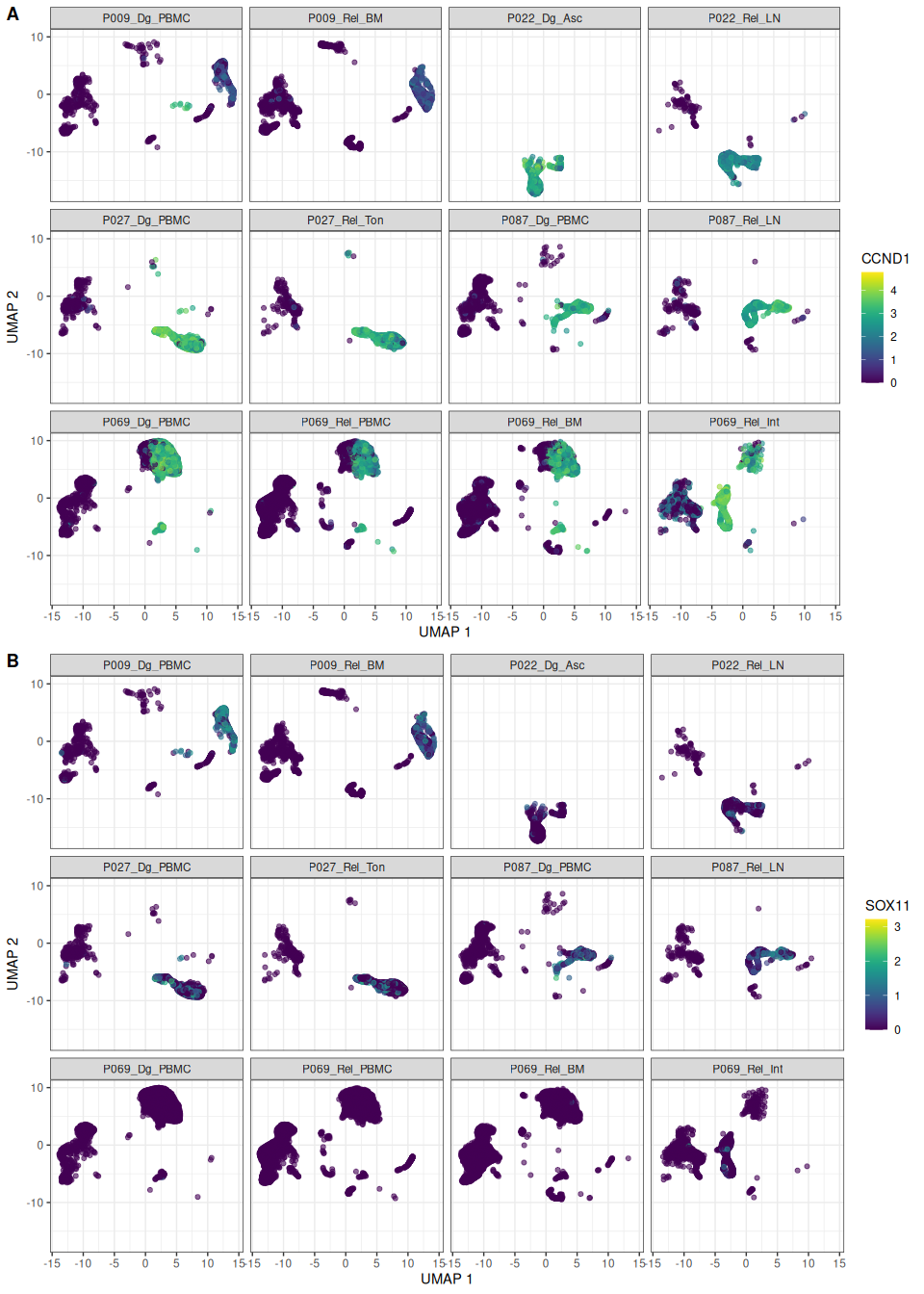


**Supplemental figure 2.** Feature UMAP plots faceted by sample colored by expression of *CCND1* (**A**) and *SOX11* (**B**). Mean of *CCND1* log counts was 0.14 in normal cells, 1 in MCL cells of P009 and 2.7 in other patients with nodal MCL. Mean of *SOX11* log counts was 0.002 in normal cells and 0.42 in MCL cells of nodal MCL patients.


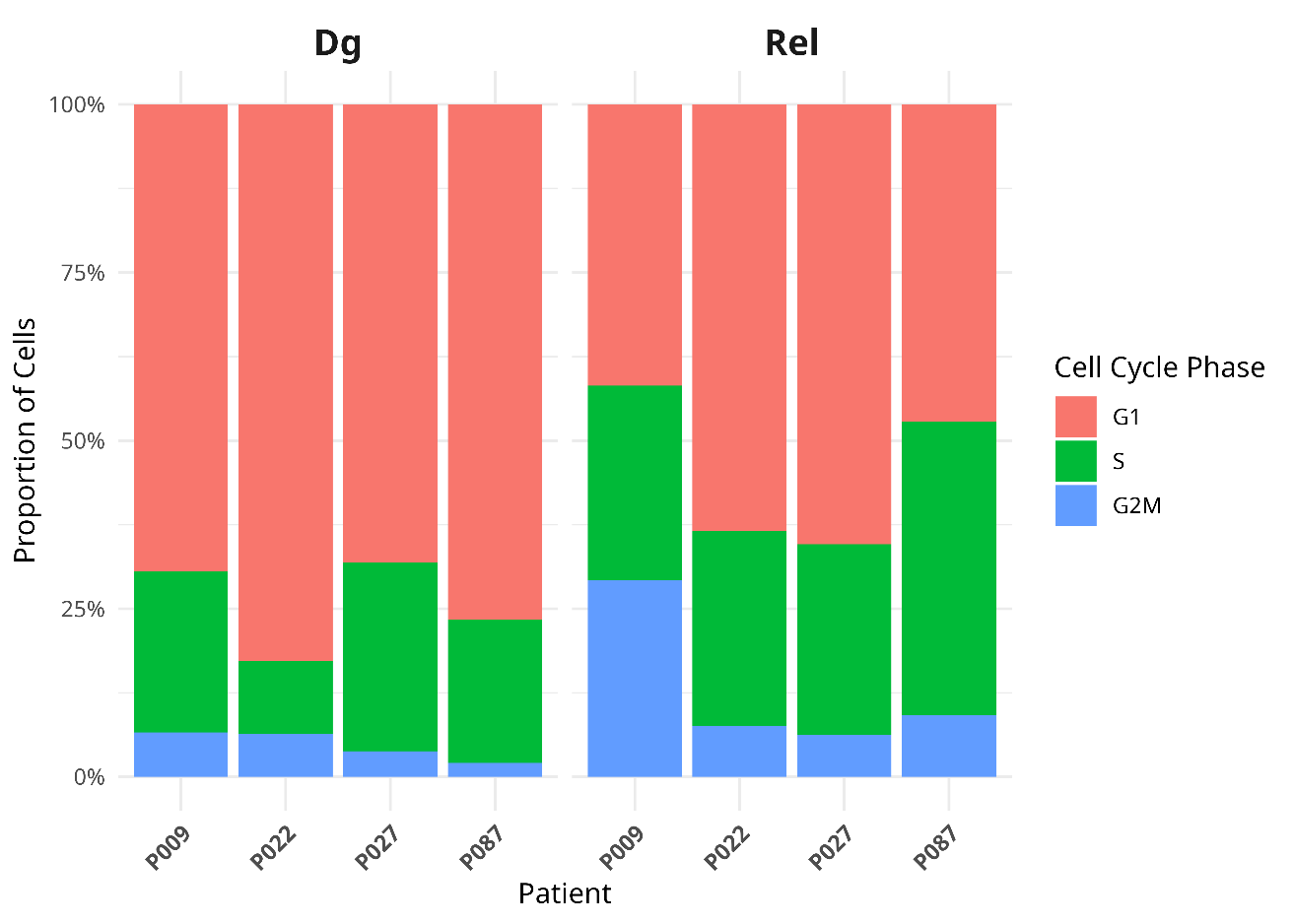


**Supplemental Figure 3. Cell cycle phase distribution across patients and timepoints.** Stacked bar chart showing the proportion of cells in each cell cycle phase (G1, S, and G2M) for five patients at diagnosis (Dg) and relapse (Rel) timepoints.


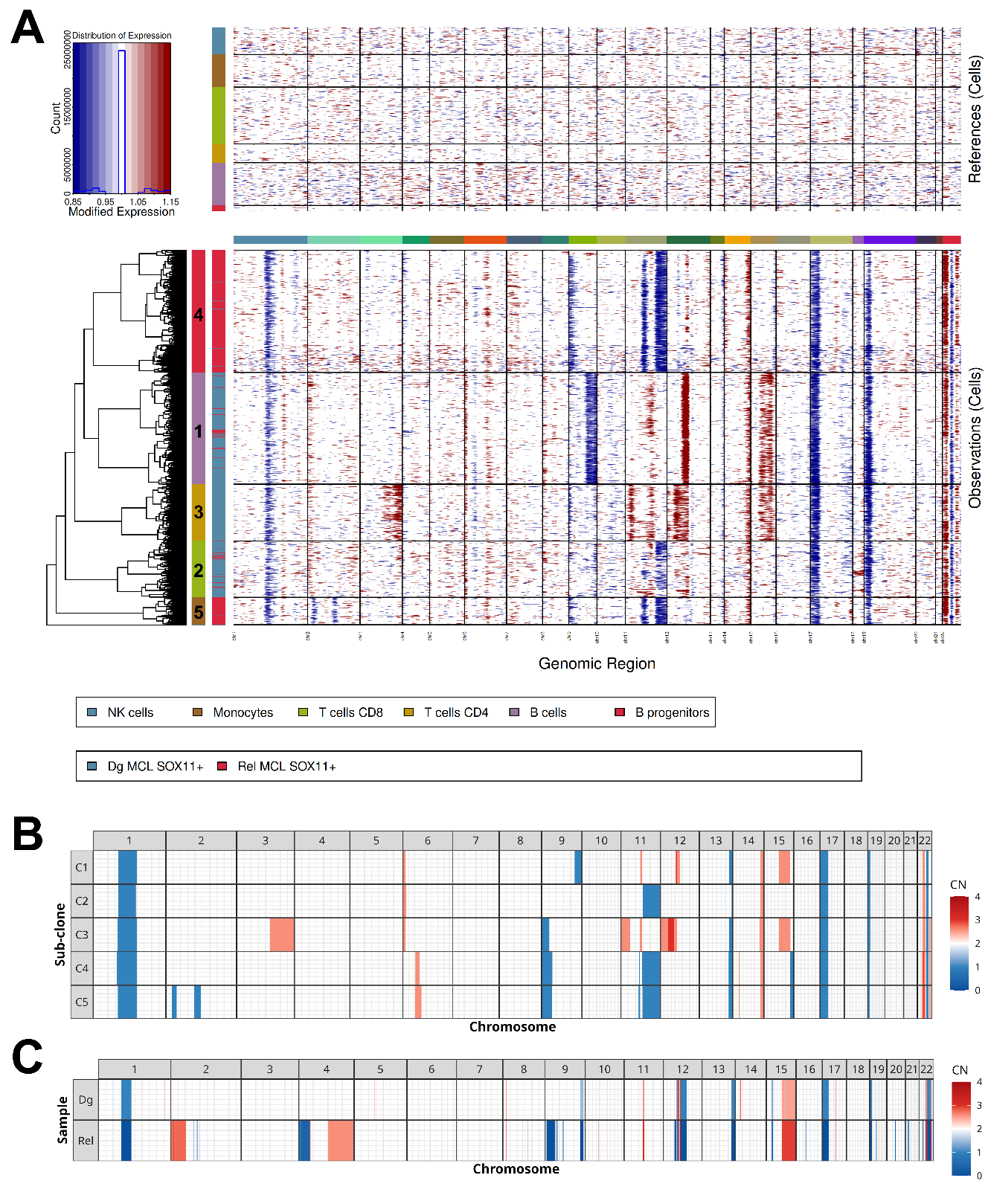


**Supplemental Figure 4.** CNV-based subclonal analysis of P009. **A:** Joint clustering of inferred CNV patterns in diagnosis (Dg) and relapse (Rel) MCL cells using inferCNV. Upper heatmap shows expression patterns of reference cells. Lower heatmap shows tumor cells. Rows represent individual cells clustered by CNV similarity, with subclone assignments indicated by the left annotation bar. Columns represent genomic regions ordered by chromosomal position. Red indicates CNV gains, blue indicates losses, and white represents diploid regions. Sample origin is indicated by the right annotation bar with legend below the heatmap (blue – diagnosis, red – relapse). **B**: Averages of CNVs inferred from scRNA-seq data per each sub-clonal cluster. **C**: CNVs predicted using WES data from Dg and Rel samples.

**
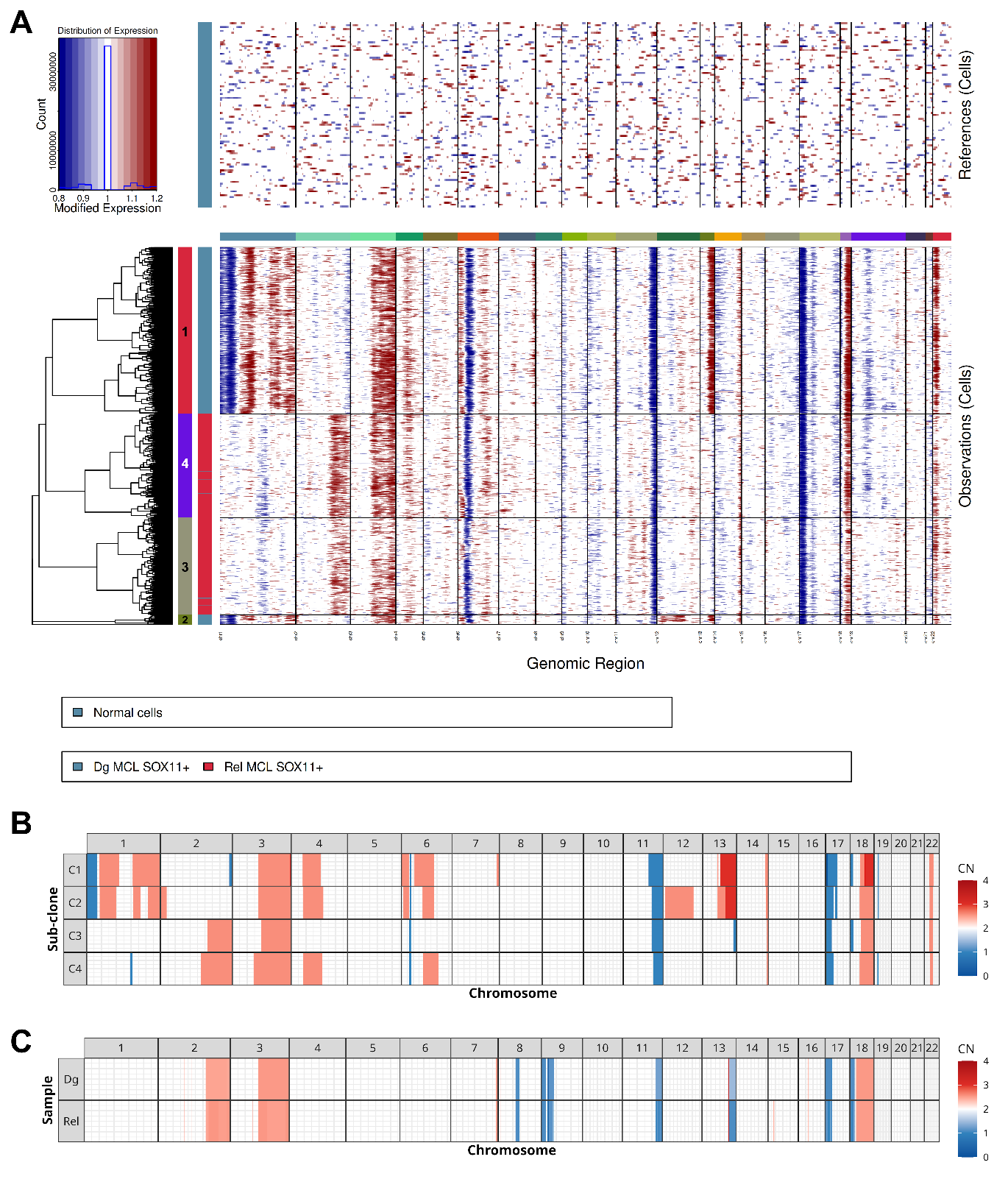
**

**Supplemental Figure 5.** CNV-based subclonal analysis of P022. **A:** Joint clustering of inferred CNV patterns in diagnosis (Dg) and relapse (Rel) MCL cells using inferCNV. Upper heatmap shows expression patterns of reference cells. Lower heatmap shows tumor cells. Rows represent individual cells clustered by CNV similarity, with subclone assignments indicated by the left annotation bar. Columns represent genomic regions ordered by chromosomal position. Red indicates CNV gains, blue indicates losses, and white represents diploid regions. Sample origin is indicated by the right annotation bar with legend below the heatmap (blue – diagnosis, red – relapse). **B**: Averages of CNVs inferred from scRNA-seq data per each sub-clonal cluster. **C**: CNVs predicted using WES data from Dg and Rel samples.

**
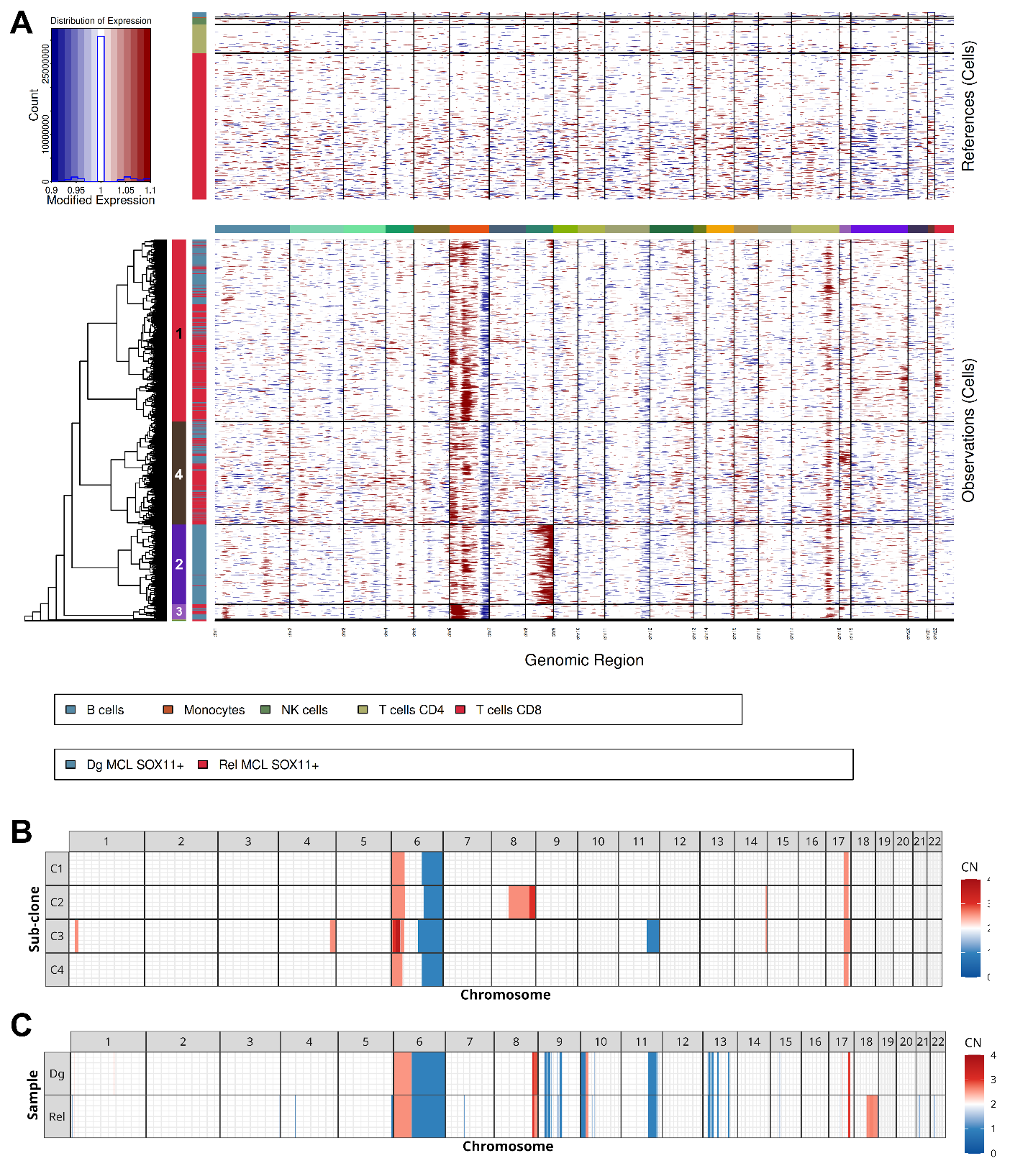
**

**Supplemental Figure 6.** CNV-based subclonal analysis of P027. **A:** Joint clustering of inferred CNV patterns in diagnosis (Dg) and relapse (Rel) MCL cells using inferCNV. Upper heatmap shows expression patterns of reference cells. Lower heatmap shows tumor cells. Rows represent individual cells clustered by CNV similarity, with subclone assignments indicated by the left annotation bar. Columns represent genomic regions ordered by chromosomal position. Red indicates CNV gains, blue indicates losses, and white represents diploid regions. Sample origin is indicated by the right annotation bar with legend below the heatmap (blue – diagnosis, red – relapse). **B**: Averages of CNVs inferred from scRNA-seq data per each sub-clonal cluster. **C**: CNVs predicted using WES data from Dg and Rel samples.

**
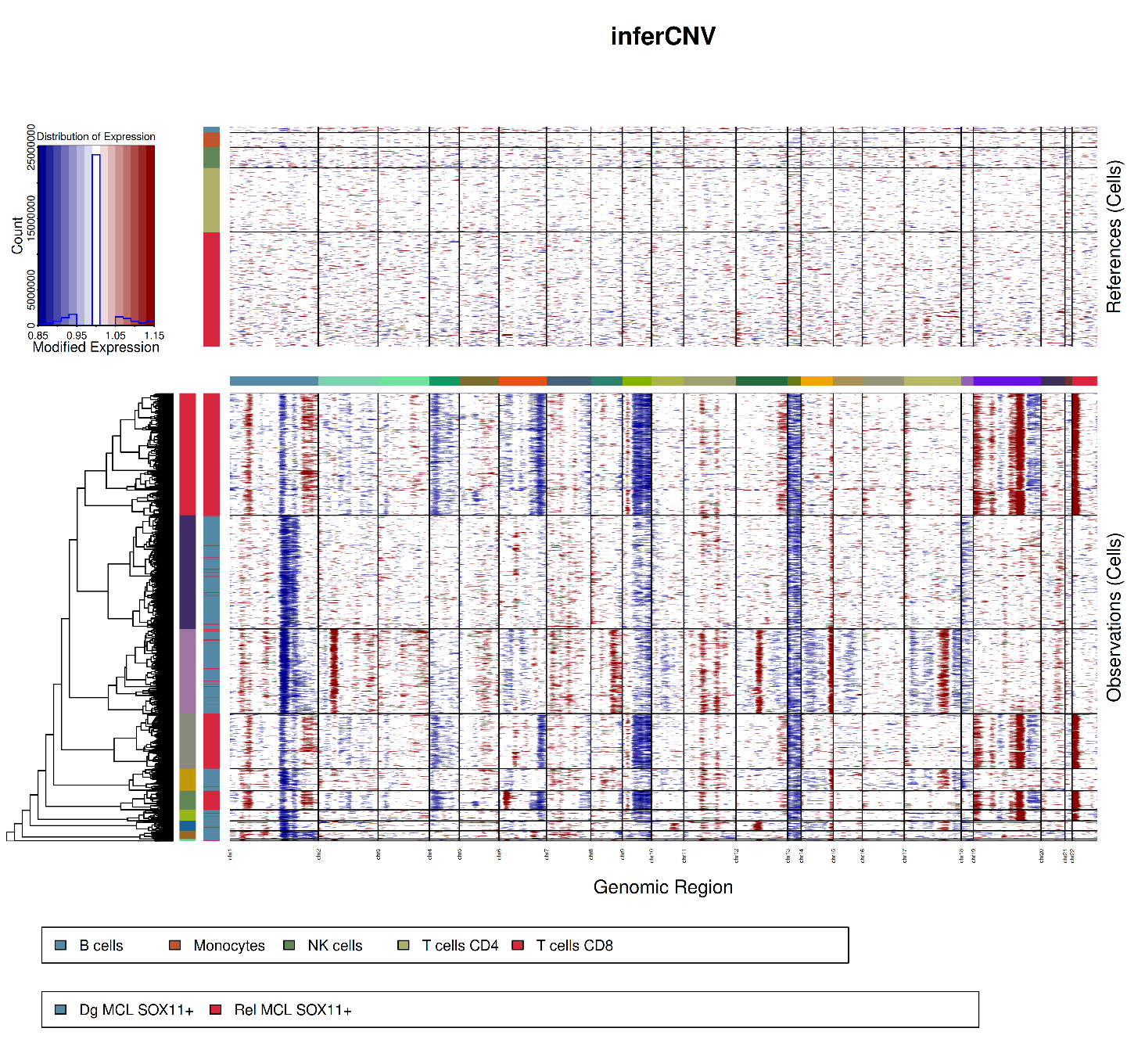
**

**Supplemental Figure 7.** CNV-based subclonal analysis of P087. Joint clustering of inferred CNV patterns in diagnosis (Dg) and relapse (Rel) MCL cells using inferCNV. Upper heatmap shows expression patterns of reference cells. Lower heatmap shows tumor cells. Rows represent individual cells clustered by CNV similarity, with subclone assignments indicated by the left annotation bar. Columns represent genomic regions ordered by chromosomal position. Red indicates CNV gains, blue indicates losses, and white represents diploid regions. Sample origin is indicated by the right annotation bar with legend below the heatmap (blue – diagnosis, red – relapse).

**
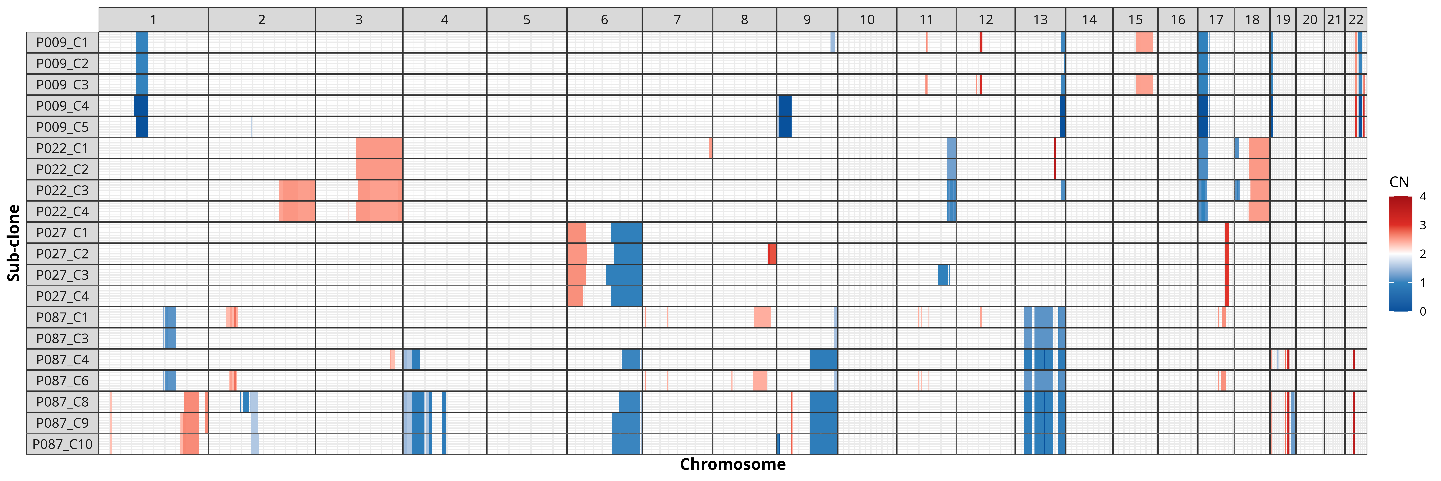
**

**Supplemental Figure 8.** Intersection of per-subclone CNVs inferred from scRNA-seq data with CNVs inferred from WES. The “therapy-sensitive” subclone CNVs were intersected with WES CNVs from Dg samples and the “therapy-resistant” subclone CNVs with WES CNVs from Rel samples. Red indicates CNV gains, blue indicates losses, and white represents diploid regions.

**
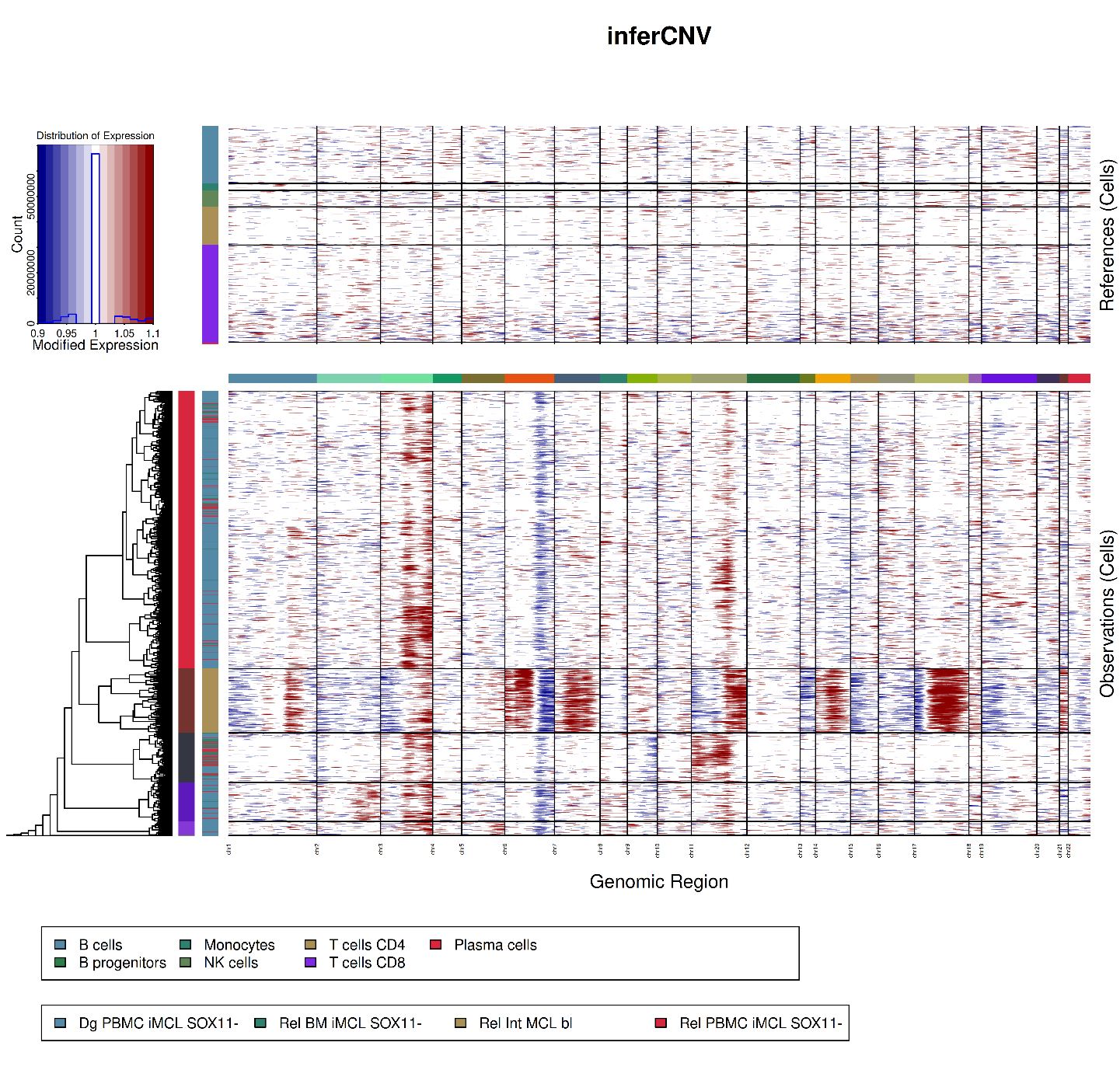
Supplemental Figure 9.** CNV-based subclonal analysis of P069. Joint clustering of inferred CNV patterns in diagnosis (Dg) from PBMC and relapse (Rel) MCL cells from PBMC, BM or intestine (Int). Upper heatmap shows expression patterns of reference cells. Lower heatmap shows tumor cells. Rows represent individual cells clustered by CNV similarity, with subclone assignments indicated by the left annotation bar. Columns represent genomic regions ordered by chromosomal position. Red indicates CNV gains, blue indicates losses, and white represents diploid regions. Sample origin is indicated by the right annotation bar with legend below the heatmap (blue – Dg, red – Rel PBMC, green – Rel BM, yellow – Rel Int).
